# CD146/sCD146 in the Pathogenesis and Monitoring of Angiogenic and Inflammatory Diseases

**DOI:** 10.3390/biomedicines8120592

**Published:** 2020-12-10

**Authors:** Xavier Heim, Ahmad Joshkon, Julien Bermudez, Richard Bachelier, Cléa Dubrou, José Boucraut, Alexandrine Foucault-Bertaud, Aurélie S. Leroyer, Francoise Dignat-George, Marcel Blot-Chabaud, Nathalie Bardin

**Affiliations:** 1 Hematology Department, Center for CardioVascular and Nutrition Research C2VN, Faculty of Pharmacy, Timone Campus, Aix-Marseille University, Institut National de la Santé et de la Recherche Médicale (INSERM), Institut National de Recherche Pour L’agriculture, L’alimentation et L’environnement (INRAE), 13005 Marseille, France; ahmadjoshkon@hotmail.com (A.J.); Julien.bermudez@ap-hm.fr (J.B.); richard.bachelier@inserm.fr (R.B.); cldubrou@gmail.com (C.D.); Alexandrine.bertaud@univ-amu.fr (A.F.-B.); Aurelie.leroyer@univ-amu.fr (A.S.L.); Francoise.dignat-george@univ-amu.fr (F.D.-G.); marcel.blot-chabaud@laposte.net (M.B.-C.); Nathalie.bardin@univ-amu.fr (N.B.); 2 Service d’immunologie, Pôle de Biologie, Hôpital de la Conception, Assistance Publique Hôpitaux de Marseille (AP-HM), 13005 Marseille, France; jose.boucraut@univ-amu.fr; 3 Pulmonology Department and Lung Transplant Team, North Hospital, Assistance Publique Hôpitaux de Marseille (AP-HM), 13015 Marseille, France; 4 Timone Neuroscience Institute, UMR CNRS 7289, Aix-Marseille University, 13005 Marseille, France; 5 Hematology and Vascular Biology Department, Hopital de la Conception, Assistance Publique Hôpitaux de Marseille (AP-HM), 13005 Marseille, France

**Keywords:** CD146, soluble CD146, inflammation angiogenesis, autoimmunity

## Abstract

CD146 is a cell adhesion molecule expressed on endothelial cells, as well as on other cells such as mesenchymal stem cells and Th17 lymphocytes. This protein also exists in a soluble form, whereby it can be detected in biological fluids, including the serum or the cerebrospinal fluid (CSF). Some studies have highlighted the significance of CD146 and its soluble form in angiogenesis and inflammation, having been shown to contribute to the pathogenesis of many inflammatory autoimmune diseases, such as systemic sclerosis, mellitus diabetes, rheumatoid arthritis, inflammatory bowel diseases, and multiple sclerosis. In this review, we will focus on how CD146 and sCD146 contribute to the pathogenesis of the aforementioned autoimmune diseases and discuss the relevance of considering it as a biomarker in these pathologies.

## 1. Introduction

CD146 is a cell adhesion molecule belonging to the immunoglobulin superfamily. It is expressed by all types of human endothelial cells, regardless of the type or caliber of the vessel, with a preferential localization at the endothelial junctions, however outside the adherents junction [1,2]. This expression is not limited to endothelial cells, as it was found to be expressed by other cell types, such as Th17 lymphocytes [3], extravillous trophoblasts [4], mesenchymal stem cells [5], melanoma cells [6], and cancer-associated fibroblasts [7]. The molecule also exists in a soluble form generated by membrane cleavage via the action of metalloproteases [8]. Soluble CD146 (sCD146) was first discovered in endothelial cell (EC) supernatants [9] and has been assayed in other biological fluids, such as cerebrospinal fluid (CSF) and blood [10,11]. In healthy subjects, sCD146 concentrations were found to be related to sex and age, with higher levels found in older males [10]. Functionally, CD146 and its soluble form are involved in angiogenesis and inflammation [12,13,14]. Indeed, CD146 participates in recruiting mononuclear cells from the vascular compartment to the site of inflammation [8,15]. During lymphocyte homing, CD146 induces cytoplasmic protrusions, which enhance lymphocyte adhesion and transmigration across the endothelium [16]. Indeed, knocking down CD146 using interfering RNA in pulmonary ECs resulted in increased cell permeability and monocyte infiltration [8]. Additionally, CD146 and its soluble form were demonstrated to mediate atherosclerotic plaque formation and progression by regulating monocyte infiltration into the arterial wall [17] and enhancing IL-8 secretion via endothelial cells (ECs), inducing neutrophils recruitment to the inflammatory site [1].

Over the past decade, some CD146 ligands have been revealed [18]. Importantly, Wnt5a, via the interaction with CD146, activates the non-canonical Wnt pathway and promotes cell mobility [19,20]. Additionally, CD146 was validated to be a co-receptor for VEGFR-2 on ECs, a function that further magnifies the angiogenic potentials of CD146 [21].

This review will primarily focus on the role of CD146 and sCD146 in the following autoimmune diseases: systemic sclerosis, diabetes mellitus, rheumatoid arthritis, inflammatory bowel disease, and multiple sclerosis. We will briefly describe the disease physiopathology and methods of diagnosis and then we will decipher how CD146 and sCD146 are implicated in the disease severity and progression. Finally, we will highlight the potential of CD146 as a biomarker or a therapeutic target.

## 2. Systemic Sclerosis

Systemic sclerosis (SSc) is a rare and severe autoimmune disease characterized by sclerosis or hardening of the skin and deep viscera. It affects the arterioles, microvessels, and connective tissues, and results in functional and structural alteration of the organs. The progression of the disease is characterized by immune dysregulation, vascular lesions, and fibrosis. However, the exact mechanism behind these phenomena are still poorly understood. At the vascular level, endothelial cells secrete a storm of pro-coagulant and pro-inflammatory factors conducive to vasospasm, leading to vessel occlusion [22]. Additionally, hypoxia and local inflammation promote fibroblast proliferation and differentiation into myofibroblasts [23]. In addition, it has been demonstrated in SSc patients that circulatory Tregs convert into IL-17A, producing T cells, which potentiate fibrotic events and drives disease progression [24]. The diagnosis of SSc is based on a combination of several clinical criteria, including skin thickness and stiffness, fingertip lesions, skin telangiectasia, and Raynaud’s phenomenon. In fact, 90% of SSc patients display antinuclear antibodies [25,26] that are directed against topoisomerase I, centromere, or RNA polymerase III, thus making them ideal molecules for disease diagnosis and prognosis. However, RNA polymerase III can also be detected in the sera of cancer patients or in patients with hypertensive kidney disease [27,28]. Unfortunately, SSc remains an incurable disease with a high mortality rate. A few clinical studies are investigating the efficiency of some drugs that may have the potential to heal the disease, however currently there is no effective treatment [29]. Thus, further investigations in the pathogenesis of this disease is required to identify new molecular targets and develop alternative therapeutic approaches.

Of importance, sCD146 concentration was significantly higher in two cohorts of SSc patients as compared to healthy subjects [30]. Moreover, the serum level of sCD146 was directly correlated with pulmonary fibrosis and digital gangrene. Patient follow-up results revealed unfavorable disease progression as sCD146 levels decrease [30]. Importantly, these findings were demonstrated by two different teams [31,32]. Additionally, in a murine model of bleomycin-induced cutaneous fibrosis, Kaspi et al. showed that CD146 plays a pivotal role in cutaneous fibrogenesis. Indeed, CD146-deficient mice (CD146KO) developed significantly more fibrosis than wild-type (WT) mice, and the injection of sCD146 in parallel with bleomycin hampered this phenomenon [30]. Interestingly, the canonical Wnt pathway was found to be dysregulated with an overactivation of β-catenin, the main Wnt profibrotic actor, in the absence of CD146 [30]. These results demonstrate a novel role of CD146 in the process of fibrosis, mediate the effects, at least in part, by regulating the canonical and non-canonical Wnt signaling. Furthermore, a positive correlation between sCD146 serum levels and IL-17A concentrations has been evidenced. Indeed, the percentage of Th17 cells expressing CD146 was higher in patients with SSc and inversely correlated with pulmonary fibrosis [32]. Additionally, an augmentation in the percentage of CD146 + Th17 cells was detected in the serum of SSc patients in a way similar to that of serum sCD146, suggesting that the increase in CD146 + Th17 cells could be the consequence of the elevated serum concentrations of sCD146.

These data confirm the physiological importance of CD146 in SSc, necessitating further investigations to prove its relevance as a molecular target in therapy.

## 3. Diabetes Mellitus

Diabetes mellitus is a clinical syndrome characterized by blood hyperglycemia higher than 7 mmol/L (1.26 g/L) under fasting conditions or 11.1 mmol/L (2 g/L) in any other conditions. Hyperglycemia is the consequence of an absolute or relative insulin deficiency. In general, three main mechanisms drive insulin resistance: (1) diminished insulin secretion by the pancreas; (2) insulin antagonists in the plasma; (3) impaired insulin response in target tissues [33]. During the course of the disease, an increase in plasma osmolarity occurs, which induces diuresis with polyuropolydypsia [34]. This clinical consequence is further worsened in cases of cardiovascular complications (diabetic microangiopathies related to hyper-glycosylation of the capillary membrane), which may manifest as strokes, kidney failure, blindness, and heart failure [35]. Moreover, hyperfiltration as a result of endothelial dysfunction is also observed in diabetic nephropathy. Importantly, hyperglycemia induces the generation of advanced glycation end-products, Reactive Oxygen Species (ROS), and pro-inflammatory cytokines [36], which lead to a decrease in NO synthesis. As a consequence, endothelial dysfunction reduces vasodilatation but increases vascular permeability, causing renal dysfunction.

In the context of diabetic nephropathy, vascular damage occurs, which elevates the serum level of sCD146. This increase in sCD146 is correlated with renal dysfunction. Moreover, serum sCD146 has been demonstrated to be a reliable biomarker for detecting renal damage with higher precision than the creatinine/albumin ratio in urine [37]. Furthermore, expression of CD146 is upregulated in kidney biopsies of patients with diabetic nephropathy as compared to normal kidney sections, mainly localized in glomerular tufts, kidney arterioles, and tubular compartments [37]. In addition, in vitro experiments on tubular epithelial cells showed that hyperglycemia potently induces CD146 expression on these cells and enhances sCD146 concentrations in the culture supernatant [38]. In fact, sCD146 serum levels correlate with diabetic nephropathy disease progression, which increases progressively until reaching stage IIIb and declines afterwards [39]. The decrease in CD146 expression can be explained by the diminution of glomerular ECs in severe diabetic nephropathy [40]. Likewise, another study showed that serum level of sCD146 positively correlates with atherosclerosis, which can be used as a biomarker for detecting disease progression and is even more effective than the currently utilized method, namely the measure of the thickness of the carotid intima-media [41]. Thus, sCD146 is a good biomarker for the early detection and management of disease complications related to diabetic micro- and macroangiopathies, especially in diabetic nephropathy, the most common complication of diabetes.

## 4. Rheumatoid Arthritis

Rheumatoid arthritis is a systemic autoimmune disease that manifests as a set of chronic polyarticular and inflammatory rheumatisms, which attack the synovial capsule and evolve through relapses [42]. The local inflammation in the articular joints recruits mononuclear cells, which in turn produce pro-inflammatory cytokines such as TNF-α, IL-1β, and IL-6 [43]. This inflammatory microenvironment aids in recruiting neutrophils, monocytes, and circulating lymphocytes, all of which exacerbate the inflammatory lesion. The methods for diagnosing rheumatoid arthritis were defined by the European League Against Rheumatism(EULAR)/American College of Rheumatology(ACR) 2010 criteria [44]. Clinically, the joints of the wrists, the metacarpophalangeal joints, and the proximal interphalangeal joints are primarily affected by the disease. In fact, joint puncture testing shows a synovial fluid rich in neutrophils and inflammatory cytokines, while in the blood compartment there is an elevation in inflammatory markers such as erythrocyte sedimentation and C-reactive protein (CRP). Additionally, rheumatoid factors were found to be present in 50% of rheumatoid arthritis patients. However, rheumatoid factor does not seem to be a specific marker for this pathology, as it can be found in other autoimmune, infectious, and lymphoproliferative diseases [45]. In addition, serum testing for cyclic citrullinated peptide (CCP) antibodies can only predict the progression of rheumatoid arthritis disease [46].

Importantly, sCD146 concentrations were found to be significantly increased in the sera of patients with rheumatoid arthritis as compared to healthy subjects [31]. Similarly, sCD146 was elevated in the joint fluid of rheumatoid arthritis patients as compared to patients with traumatic joint injury or non-rheumatoid arthritis polyarthritis [47]. Importantly, the sCD146 concentration positively correlates with the extent of joint stiffness and the number of swollen joints in rheumatoid arthritis patients [47]. In addition, during early phases of the disease (<1 year after diagnosis), sCD146 serum concentration was shown to be elevated, suggesting a possible role of CD146 in the establishment of inflammatory and angiogenic responses. Furthermore, the number of Th17 cells secreting IL-17A and IL-22 does not appear to be significantly increased in the blood of rheumatoid arthritis patients, contrary to results found for groups with spondyloarthropathy [48]. Finally, a study has shown that CD146 in the synovial fluid is involved in mediating inflammation by recruiting circulating lymphocytes via the interaction with their surface CD146 [49]. Additionally, sCD146 may also be involved in the assessment of disease severity in its early phase in the absence of other biological elements, such as anti-CCP and anti-RF antibodies. The role of CD146 in the maintenance of inflammatory response requires further studies to determine if it could be a good target for the disease.

## 5. Inflammatory Bowel Disease

Inflammatory Bowel Disease (IBD) includes two pathologies causing inflammation of the wall of the digestive tract: Crohn’s disease and ulcerative colitis. In Crohn’s disease, the inflammation can be localized in any spart of the digestive system, but most often affects the ileum and colon (ileo-cecal involvement), and to a lower degree the region of the anus. In ulcerative colitis, it is localized in the rectum and extends continuously towards the cecum without reaching the small intestine. At the origin of these diseases, an imbalance in the composition of the intestinal flora creates an active terrain for macrophages [50,51]. These immune cells become activated by bacterial lipopolysaccharides and synthesize large amounts of IL-12 and TNF-α [52]. TNF-α acts as a hallmark in the initiation of the disease [53] by recruiting other innate immune cells, sustaining pro-inflammatory cytokine synthesis, increasing intestinal permeability, promoting tissue destruction, and providing survival signals to activated T cells. Indeed, the key role of TNF-α in inducing mucosal inflammation is attested by the therapeutic efficacy of anti-TNFs [54]. Other therapies directed against inflammatory cytokines, such as anti-IL-12 and anti-IL-22 [54], or those impeding or involving lymphocyte trafficking (such as vedolizumab) [55] are efficient in hampering disease progression. At present, there is a scarcity of biomarkers available for diagnosing IBD, with the only biomarker being the anti-Saccharomyces cerevisiae antibodies (ASCA) for Crohn’s disease and anti-neutrophil cytoplasmic antibodies (ANCA) for ulcerative colitis. Besides, these biomarkers often fail to adequately diagnose patients, as some IBD patients are antineutrophil cytoplasmic antibodies (ANCA)-negative and ASCA-negative. On the other hand, calprotectin, an inflammatory marker, is often measured directly in the stool, however this test is used to evaluate the effectiveness of treatment.

CD146 staining on mucosal biopsies from patients with Crohn’s disease or ulcerative colitis showed an increased expression of CD146 in the active disease area of the intestine compared to a healthy control group [56,57]. Additionally, sCD146 is decreased in the sera of patients with Crohn’s disease during the active disease phase while soluble CD31 decreases only during the relapses of ulcerative colitis. Of interest, anti-TNF-α therapy restored sCD146 to physiological levels along with an overall clinical improvement in Crohn’s disease [58]. Furthermore, in a model of murine colitis, the conditional knock-out for endothelial CD146 reduced lymphocyte infiltration and epithelial inflammation as compared to WT mice. Likewise, blocking endothelial CD146 using anti-CD146 antibody (AA98) also decreased inflammation and disease severity in murine models of colitis [59]. Moreover, clinical data have shown some patients receiving anti-TNF-α therapy become refractive to the treatment [60]. Thus, CD146 represents a potential target in IBD therapy that can be combined with other anti-inflammatory biotherapies.

## 6. Multiple Sclerosis

Multiple sclerosis is an inflammatory autoimmune disease targeting the central nervous system (CNS) and characterized by demyelinating plaques that gradually develop into sclerotic lesions. The pathophysiology of the disease involves the mobilization of T-cells, monocytes, and macrophages from the periphery to the central nervous system. This will create an inflammatory microenvironment rich in cytokines and autoantibodies that will target and demyelinate neuronal axons. This disturbs the communication between consecutive neurons and consequently impairs the speed of propagation of the nerve impulse. Indeed, the loss of axons occurring during the acute inflammatory phase of the disease explains the permanent disability [61]. The diagnosis is based on a set of criteria as defined by McDonald and updated in 2010 by Polman [62]. The inflammatory process is localized in the central nervous system, as evidenced by the presence of oligoclonal bands in the CSF. Natalizumab, a monoclonal antibody targeting and blocking α4 integrin, is commonly used in therapy to inhibits lymphocyte entry into the CNS [63]. Unfortunately, this type of therapy hampers the immunity in the CNS, making patients more prone to viral infections [64].

Among the various immune cells, CD146 is preferentially expressed on a subset of activated T cells, the Th17 lymphocytes [65]. This population of cells is primarily found in the CNS, whereby CD146 was found to be a key adhesion molecule mediating this localization. Indeed, in vivo and ex vivo experiments demonstrated that the blocking of CD146 on T-lymphocytes potently reduced their migratory capabilities [66]. The recruitment of Th17 lymphocytes involves interactions with the endothelium in a mechanism that is dependent on PSGL-1, but not VLA-4 [67]. Interestingly, CD146 expression becomes upregulated on T cells following the treatment with the anti-VLA4 antibody natalizumab, suggesting that CD146 may provide an escape mechanism [68]. CD146 is, therefore, believed to play a key role in the extravasation and trafficking of immune cells. This was confirmed by Duan et al., who showed in an experimental acute encephalitis model that endothelial CD146 was necessary for the diapedesis of immune cells into the nervous system [69]. This transmigration is possible because the interaction between CD146 and laminin 411 allows lymphocyte entry through the choroid plexus [70]. In addition, sCD146 levels increase in the CSF of patients with active versus inactive MS. These levels correlate with the loss of integrity of the blood-brain barrier and the presence of pro-inflammatory cytokines such as TNF-α, IFN-γ, IL-2, and IL-17A [11]. CD146, thus, constitutes an important target in multiple sclerosis therapy it can be combined with other drugs such as natalizumab to overcome the developed resistance.

## 7. Interest of sCD146 and CD146 in Autoimmune Diseases

Owing to its angiogenic and inflammatory effects, CD146 represents an appealing target in autoimmune diseases. In this review, we highlight the impacts of CD146 and sCD146 on the pathophysiology of autoimmune disorders and emphasize their potential as prognostic biomarkers or therapeutic targets. A summary of the results is presented in Table 1. We report that sCD146 was detectable in various biological fluids such as blood [71], CSF [11], synovial fluid [47], and cell culture supernatant [9]. Depending on the pathology, the sCD146 concentration may fluctuate according to the disease prognosis. For example, as in systemic sclerosis, serum sCD146 decreases as the clinical manifestation of the disease worsens. Similarly, a decrease in serum sCD146 concentration was validated during IBD relapse. However, in multiple sclerosis, the sCD146 concentration in the CSF increases during the relapse phase [11]. In light of these results, we can conclude that sCD146 is not a specific diagnostic marker. Indeed, it is increased in several pathologies and to date has not been associated with any particular clinical form of disease. In idiopathic inflammatory myopathies, it may, in association with other soluble (sPECAM-1, sICAM1) and membrane (CD146, ICAM1, CD31) markers, contribute to their classification [72].

However, sCD146 seems to be an appealing marker for disease monitoring. For instance, in systemic sclerosis (SSc), for which no marker to date is predictive of progression, lower sCD146 concentrations in patients with SSc are associated with greater pulmonary complications and digital gangrene [30,31].

**Figure 1:**
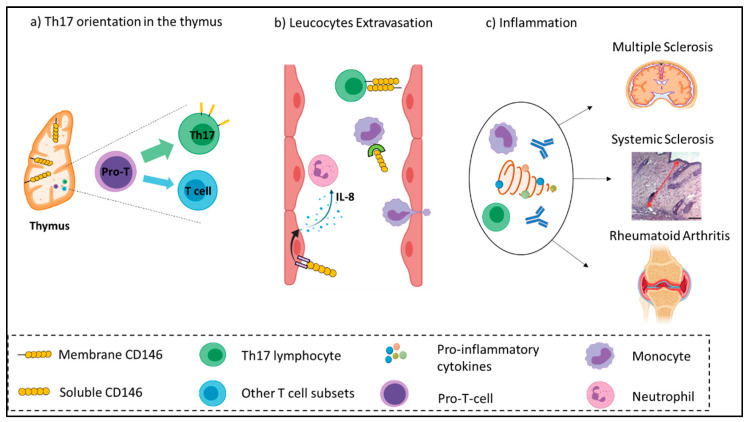
Illustration summarizing the inflammatory role of CD146 in autoimmune diseases. (**a**) CD146 supports thymic development and architecture and promotes the differentiation of naïve T cells from the Th17 subset. (**b**) In the blood compartment, CD146 enhances the adhesion, rolling, and extravasation of lymphocytes and monocytes across the endothelium. The soluble form of CD146 favorizes IL-8 secretion by endothelial cells, which in turn recruit neutrophils. (**c**) Following extravasation, immune effectors produce a storm of inflammatory cytokines, which exacerbate inflammation and subsequently damage target organs such as the Central Nervous System (CNS), skin, and joints.

How to explain the relevant role of CD146 in autoimmune diseases? Different hypotheses can be put forward to explain the involvement of CD146/sCD146 in autoimmune pathology, such as its vascular expression, its role in Th17 orientation or in inflammation (Figure 1).

Due to its constitutive expression on the vascular endothelium, CD146 is strongly represented in the organism, explaining the relatively high serum concentrations in the range of 300 ng/mL [71], while that of VEGF is close to pg/mL.

In addition, CD146 plays an essential role in the maintenance of the thymic architecture and functions by mediating lymphocyte transmigration, allowing the migration and trafficking of lymphocytes to the secondary lymphoid organs [73]. While in physiological condition 2% of T lymphocytes express CD146, this condition is notably associated with the orientation of Th17 cells towards the function of the memory effector lymphocyte [74,75]. Of importance, the role of Th17 lymphocytes in autoimmune diseases has only recently been demonstrated. Th17 lymphocyte cytokines allow the recruitment and activation of neutrophils, monocytes, and polynuclear cells, which in turn synthesize numerous pro-inflammatory mediators, such as TNF-α, IL-1β, IL-6, GM-CSF, and metalloproteases.

The implication of CD146 in Th17 cell differentiation from the T cell precursor may be associated with the regulatory role of CD146 in the Wnt pathway. Indeed, in the presence of membrane CD146, these cells shift towards a phenotype associated with the non-canonical Wnt pathway [19], resulting in an increase in Th1 and Th17 cells and an increase in neuro-inflammation phenomena [76], as the canonical Wnt pathway inhibits differentiation into Th17 lymphocytes.

The functions of CD146 on migration, proliferation, and inflammation allow us to better understand its involvement in autoimmune diseases. Indeed, CD146 could be implicated in the recruitment of activated lymphocytes at the site of inflammation and in the extravasation of activated T-lymphocytes [49]. This phenomenon could be explained by CD146-mediated induction of microvilli [16], which promote the rolling of lymphocytes via their interaction with markers of inflammation, such as VCAM-1 and CD146 binding to the extracellular matrix protein laminin 411 [77]. It has been described that CD146 can bind with the non-canonical Wnt pathway ligand Wnt5a to form structures called Wnt5a-receptor-actin-myosin-polarity, which enhance cell migration functions [20].

In addition to Th17 lymphocytes, CD146 is also involved in the recruitment of inflammatory monocytes [8]. Soluble CD146, by binding to inflammatory monocytes [78], may promote the pathogenic action of autoantibodies through an ADCC mechanism associated with monocyte recruitment.

## 8. Interest in CD146 and sCD146 as Molecular Targets

The development of therapeutic monoclonal antibodies benefits from the progress made in the field of biotechnology. However, the number of antigens that can be targeted by monoclonal antibodies is potentially infinite. Hence, there is a need to define the therapeutic targets. Based on clinical and pathophysiological data, CD146 and sCD146 are interesting therapeutic targets in some pathologies, in particular systemic sclerosis and multiple sclerosis. Thus, the development of an antibody directed against CD146 or sCD146 is of interest, especially for treating several autoimmune pathologies, in particular those where there are no etiological therapies, such as systemic sclerosis. Zhang et al. used an anti-CD146 antibody that blocks the interaction between CD146 and the Wnt pathway in a model of bleomycin-induced cutaneous sclerosis and decreases the effects on cutaneous fibrosis [79]. In multiple sclerosis, endothelial CD146 blockade decreases the passage of lymphocytes into the central nervous system in a mouse model of multiple sclerosis [70]. Targeting CD146 is an attractive alternative or complementary therapy to the existing therapies. The newly developed anti-sCD146 antibody M2J-1 [80] can be employed to inhibit IBD relapse or rheumatoid arthritis, however this requires further investigation.

Finally, we can also imagine that administration of soluble recombinant molecules such as sCD146 in systemic sclerosis could represent a new therapy for the management of the disease if we can confirm the effects observed by Kaspi et al. [30] and that the molecule has no side effects in humans. Soluble EGF-like domain-containing protein 7, an extracellular protein, seems to act in multiple sclerosis by inhibiting CD146 expression on endothelial cells, and consequently lymphocyte trafficking to central nervous system [81].

## Figures and Tables

**Table 1:** CD146 and soluble CD146 in immune diseases.

Pathology	Membrane CD146			Soluble CD146		
	Cell Population	Function	Ref	Fluid of Interest	Role	Ref
Systemic sclerosis	Fibroblasts	Protects from fibrosis	Kaspi et al. [30]	Serum	Follow-up	Kaspi et al. [30]
	Th17 cells	Migration	Gabsi et al. [32]			
Diabetes mellitus	Tubular epithelial cells	Not determined	Wang et al. [38]	Serum	Follow-up	Saito et al. [39]
Rheumatoid arthritis	Lymphocytes	Migration	Pickl et al. [49]	Synovial fluid	Prognosis	Neidhart et al. [47]
Inflammatory Bowel Disease	Endothelial cells	Not determined	Bardin et al. [56]	Serum	Follow-up	Bardin et al. [56]
Multiple sclerosis	Th17 cells	Migration	Breuer et al. [70]	CSF	Not determined	Duan et al. [11]
